# Education and Genetic Risk Modulate Hippocampal Structure in Alzheimer’s Disease

**DOI:** 10.14336/AD.2016.0305

**Published:** 2016-10-01

**Authors:** Johanna Baumgaertel, Robert Haussmann, Antonia Gruschwitz, Annett Werner, Antje Osterrath, Jan Lange, Katharina L. Donix, Jennifer Linn, Markus Donix

**Affiliations:** ^1^Department of Psychiatry, University Hospital Carl Gustav Carus, Technische Universität Dresden, 01307 Dresden, Germany; ^2^DZNE, German Center for Neurodegenerative Diseases, Dresden, Germany; ^3^Department of Neuroradiology, University Hospital Carl Gustav Carus, Technische Universität Dresden, 01307 Dresden, Germany

**Keywords:** hippocampus, apolipoprotein E, cognitive reserve, education

## Abstract

Genetic and environmental protective factors and risks modulate brain structure and function in neurodegenerative diseases and their preclinical stages. We wanted to investigate whether the years of formal education, a proxy measure for cognitive reserve, would influence hippocampal structure in Alzheimer’s disease patients, and whether apolipoprotein Eε4 (APOE4) carrier status and a first-degree family history of the disease would change a possible association. Fifty-eight Alzheimer’s disease patients underwent 3T magnetic resonance imaging. We applied a cortical unfolding approach to investigate individual subregions of the medial temporal lobe. Among patients homozygous for the APOE4 genotype or carrying both APOE4 and family history risks, lower education was associated with a thinner cortex in multiple medial temporal regions, including the hippocampus. Our data suggest that the years of formal education and genetic risks interact in their influence on hippocampal structure in Alzheimer’s disease patients.

In Alzheimer’s disease, the most common type of dementia [1], pathological brain changes appear many years before the onset of clinical symptoms [2]. It is the interplay of risks and protective factors that informs research focusing on Alzheimer’s disease prevention, or the characteristics and time course of its subclinical stages preceding dementia. Heritability for late-onset Alzheimer’s disease is about 60-80% [3], and may decrease with higher age of disease onset [4]. This is consistent with data suggesting increasing importance of environmental factors in Alzheimer’s disease development during the life span [5].

The cognitive reserve hypothesis implies that age- or disease-related brain changes can be tolerated without obvious clinical symptoms [6], either through protective mechanisms, for example a greater neural capacity less susceptible to damage, or the recruitment of compensatory neural networks [7]. The years of formal education represent a proxy measure for cognitive reserve (for review see [8]). Data from older people living under uniform environmental and occupational conditions support this approach, the authors found a strong association between education and dementia [9]. The protective value of education for the incidence and prevalence of dementia and Alzheimer’s disease has been demonstrated consistently [8, 9]. Functional neuroimaging studies show that higher education is associated with a slower rate of beta-amyloid deposits and preserved brain glucose metabolism [10]. Structural brain imaging data also suggest an influence of educational status on cortical thickness or volumetric measures, although data vary depending on the brain regions investigated and the presence of cognitive impairment among study participants [11-13]. Sole-Padulles and colleagues found larger brain volumes in healthy older people with high cognitive reserve [12], Serra and colleagues detected reduced regional gray matter volumes in the supramarginal gyrus and in the right posterior cingulate gyrus among lower educated people with mild cognitive impairment and Alzheimer’s disease [13]. However, the authors also highlight that higher education is not associated with greater gray matter volume in all brain regions, and that functional MRI data are useful to additionally characterize cognitive reserve capacity [12, 13].

The ε4 allele of the apolipoprotein E gene (APOE4) is the most important genetic risk factor for Alzheimer’s disease, associated with an approximately eight-fold increased risk for developing the disease, and a more than 15 years earlier disease onset among homozygous carriers [14]. APOE4 is associated with structural and functional brain changes in medial temporal lobe regions affected early by beta-amyloid plaques and neurofibrillary tangle burden, such as the entorhinal cortex and the hippocampus [15, 16]. The APOE4 allele is associated with reduced cortical thickness in medial temporal lobe regions, specifically in the entorhinal cortex and in the subiculum [17]. In order to investigate the influence of other and not yet detected genetic risks for Alzheimer’s disease, a first-degree family history of Alzheimer’s disease can be utilized as a composite risk factor for the complex polygenic disorder [18]. Family history risk and APOE4 are associated with independent and sometimes additive effects on disease risk, cognitive performance and neuroimaging characteristics [18, 19]. We previously showed APOE4-independent cortical thinning in medial temporal lobe regions associated with a family history of Alzheimer’s disease [20].

It is likely that genetic risks interact with protective factors such as education. Vermeiren and colleagues [21] revealed that APOE4 predicted the onset of Alzheimer’s disease only in middle- and high-educated subjects. However, it remains unknown if a possible interaction is associated with changes in local brain morphology. We hypothesized that the years of formal education, a proxy measure for cognitive reserve, and genetic risks would interact in their influence on subregional hippocampal cortical thickness in Alzheimer’s disease patients. In order to reveal possible changes in cortical thickness within individual subregions of the hippocampus and adjacent medial temporal lobe, we utilized high-resolution magnetic resonance imaging (MRI) and a computational cortical unfolding approach [22].

## MATERIALS AND METHODS

### Patients

Fifty-eight patients with Alzheimer’s disease (mean age 72.7 years ± 6.7 years) were recruited through our university hospital’s memory clinic. These patients were selected from a pool of 130 subjects varying in cognitive abilities who participated in neuropsychological examinations and MRI scanning aimed at investigating Alzheimer’s disease risk factors. After complete description of the study to the subjects, written informed consent was obtained; the university’s ethics committee approved the study (Ethikkommission der Technischen Universität Dresden, EK 41022014). Procedures were also in accord with the Helsinki Declaration of 1975. We only recruited Alzheimer’s disease patients with mild impairments (mean Mini Mental State Exam [23] score 22.7 ± 5.0) who had the cognitive capacity to consent. An independent psychiatrist established the diagnoses and evaluated the capacity to consent. Alzheimer’s disease patients met standard clinical criteria [24]. All patients underwent clinical and neuropsychological examinations, laboratory testing including APOE genotyping, and brain imaging. Four patients did not receive the MRI sequence necessary for cortical unfolding analyses (withdrew consent). The patients did not have psychiatric or neurological disorders other than Alzheimer’s disease, no cerebrovascular disease that could be associated with vascular or mixed type dementia, or any systemic disease possibly affecting brain function. All patients were on stable (> 6 months) medication with an acetyl-cholinesterase inhibitor.

Education was defined as the years of formal education up to an eventual vocational training graduation or university graduation. Our patients showed education times from 9 to 21 years (mean: 13.6 ± 2.8). All patients with an educational level below or above average were allocated to a lower or higher education group, respectively. In our sample we had 3 patients with the APOE2/3 genotype, 18 patients homozygous for the APOE3 allele, and 37 APOE4 allele carriers including 10 patients with the homozygous variant. There were no subjects with the APOE2/2 or APOE2/4 genotype. A first-degree family history of Alzheimer’s disease could be observed in 22 of our patients. Family history risk was considered positive if at least one first-degree relative had been diagnosed with Alzheimer’s disease [24]. All subjects with this risk factor had parental family history. For all patients, we defined three individual risk factor levels: The absence of APOE4 or a first-degree family history of Alzheimer’s disease (low risk), APOE4 heterozygosis or a positive family history (intermediate risk), and APOE4 homozygosis or both APOE4 and family history risk (high risk).

### Procedures

**Figure 1.**
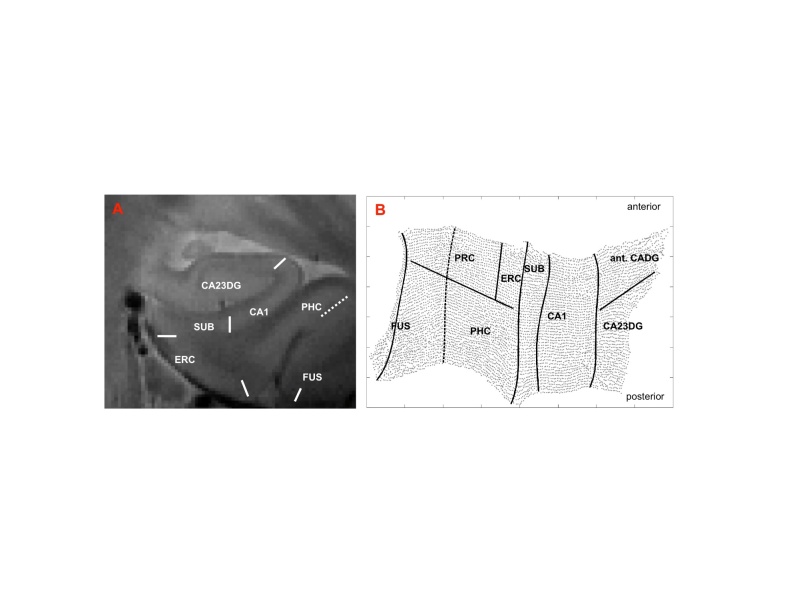
**Cortical unfolding.** After oblique coronal MRI scanning and manual segmentation of white matter and CSF on the T2 weighted MRI sequence, the resulting gray matter volume is computationally unfolded and flattened based on metric multidimensional scaling [right hemispheric flatmap shown, B]. Boundaries between the subregions are delineated on the original high-resolution MRI sequence (A) and later mathematically projected to flat map space. CA23DG=cornu ammonis fields 2,3 and dentate gyrus (the anterior part of the cornu ammonis fields and dentate gyrus [ant. CADG] is part of the CA23DG region), CA1=CA field 1, SUB=subiculum, ERC=entorhinal cortex, PRC=perirhinal cortex, PHC=parahippocampal cortex, FUS=fusiform cortex (fusiform boundary depicts the medial fusiform vertex).

MRI scanning was performed on a GE Signa HDxt (General Electric Health Care, Waukesha, Wisconsin) 3T whole brain MRI scanner. We acquired a high-resolution oblique coronal T2 weighted fast spin echo sequence [repetition time 5200ms, echo time 105ms, slice thickness 3mm, spacing 0mm, 19 slices, in-plane voxel size 0.39 x 0.39 mm, field of view 200mm], and performed a detailed analysis of hippocampal and medial temporal lobe subregions by utilizing a cortical unfolding approach [22, 25, 26]. This technique enhances the visibility of the small and convoluted structures by flattening the region’s gray matter into two-dimensional space (Figure 1). Using MRI data with very high in-plane resolution, white matter and cerebrospinal fluid are manually defined (masked) in a fist step of the procedure. Labelling pixels as white matter or cerebrospinal fluid is performed with mrGray software [27] based on changes in signal intensity values. After this segmentation, the images (white matter and cerebrospinal fluid masks, resulting gray matter) are interpolated by a factor of 7 to achieve nearly isotropic voxels of approx. 0.4mm^3^. The entire gray matter volume is then grown out in connected layers using a region-expansion algorithm, containing cornu ammonis (CA) fields 1-3 and the dentate gyrus, subiculum, entorhinal, perirhinal, and parahippocampal cortices, and the fusiform gyrus. After the computational unfolding, which is based on metric multidimensional scaling, boundaries between subfields are mathematically projected to their flat map space coordinates after their delineation on the original MRI sequence using histological and MRI atlases [28, 29]. The accuracy of the cortical unfolding technique has been previously demonstrated in different studies [17, 22, 25]. The cortical thickness of all subregions is calculated in three-dimensional space. For each gray matter voxel, the distance to the closest non-gray matter voxel is computed. In two-dimensional space, for each voxel, the maximum distance value of the corresponding three-dimensional voxels across all layers is taken and multiplied by two. Thickness in each subregion is calculated by averaging the thickness of all two-dimensional voxels. For technological details of the cortical unfolding technique see [25, 26] as well as [17] for cortical thickness measurements. In line with our previous studies applying cortical unfolding we report raw (uncorrected) data, which is the preferred strategy for investigating cortical thickness in contrast to volume [30]. Investigators performing segmentations and cortical unfolding were unaware of the patients’ clinical or demographic characteristics.

### Statistical analyses

In order to determine the influence of educational status and individual dementia risk factors on subregional hippocampal and medial temporal cortical thickness we first estimated mixed general linear models, with averaged (across subregions) thickness as the dependent variable and education status (lower/higher) and risk factor status (low/intermediate/high) as between-group factors and age and gender as covariates. We also investigated a possible interaction between education and risk factor status. After we established significance with the multivariate F tests we conducted post hoc univariate tests including linear regression analyses to determine the influence of education on regional cortical thickness among patients differing in risk factor status. Gender and

APOE allele distribution were compared with Chi-Square tests. Statistical analyses used a significance level of p<0.05.

## RESULTS

Patients with higher or lower education did not significantly differ in age or gender distribution. The frequency of the APOE4 risk allele or the presence of the family history risk factor was also comparable between the groups (Table 1).

**Table 1:** Demographic characteristics and neuropsychological scores.

Characteristics and Measures	Lower education	SD	Higher education	SD	Significance (*p*-value)^*^
**N**	32		26		
**Age** (years)	71.8	± 7.0	73.8	±6.3	*0.25*
**Female sex** (no.)	18		9		0.1
**Education** (years)	11.5	± 1.2	16.2	±1.7	**< 0.001**
**APOE status** (no.)					
2,3	2		1		*0.68*
3,3	12		6		*0.24*
3,4	13		14		*0.32*
4,4	5		5		*0.72*
**First degree family history of AD** (no.)	9		13		*0.09*
**MMSE** (score range 0-30)	21.1	± 5.2	24.7	± 4.2	**0.033**

MMSE: Mini Mental State Examination;

* Chi-Square tests for Gender and APOE status

**Figure 2.**
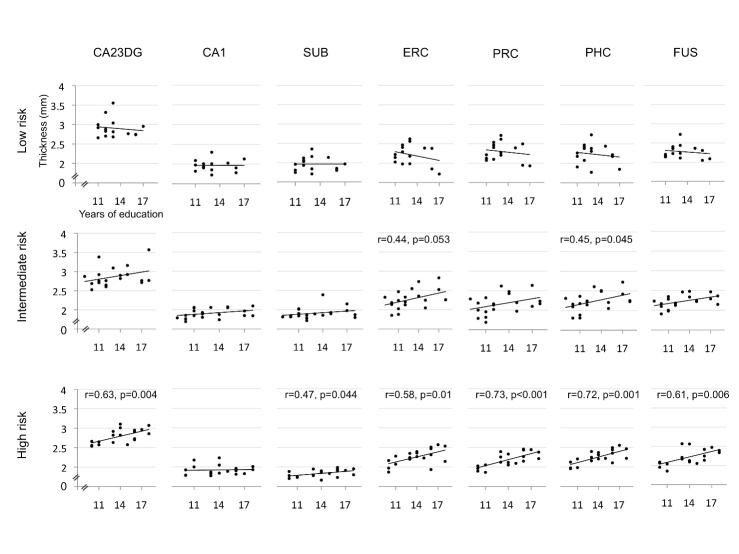
**Correlation of education and cortical thickness.** The figure illustrates the association of the years of education and cortical thickness across medial temporal subregions (legend see Figure 1). Patients homozygous for the APOE4 allele or APOE4 carriers with a first-degree family history of Alzheimer’s disease showed a positive correlation of education and thickness in all regions except CA1. Patients with an intermediate risk profile (APOE4 heterozygosis or family history risk) showed this correlation only in PHC with trends for other regions, such as the ERC. Patients in the low risk group (no APOE4 allele and no family history) did not show an association between education and thickness.

Mean cortical thickness (averaged across all subregions) did not significantly differ between higher and lower educated patients (higher educated: 2,28 mm ± 0.12 mm, lower educated: 2,20 mm ± 0.17 mm). The mixed general linear models did not show main effects for education status and risk factor status, but revealed an interaction between both factors (F=3.97, df=2,46, p=0.026). Main effects for age and gender were not significant. Post hoc analyses (Figure 2) showed no correlation between the years of education and cortical thickness in all medial temporal lobe subregions among Alzheimer’s disease patients carrying no genetic risk factors. In patients carrying either the APOE4 allele or having a first-degree family history of Alzheimer’s disease, years of education and cortical thickness were positively correlated in the parahippocampal cortex (Pearson’s r=0.45, p=0.045), and across all medial temporal subregions combined (r=0.48, p=0.031). The entorhinal cortex failed to reach significance (r=0.44, p=0.053). Among patients homozygous for the APOE4 allele or carrying both APOE4 and family history risk factors, years of education and cortical thickness were positively correlated in the hippocampus CA23DG region (r=0.63, p=0.004), subiculum (r=0.47, p=0.044), entorhinal cortex (r=0.58, p=0.01), perirhinal cortex (r=0.73, p<0.001), parahippocampal cortex (r=0.72, p=0.001), fusiform gyrus (r=0.61, p=0.006), and across all subregions combined (r=0.77, p<0.001).

## DISCUSSION

We demonstrate an interaction between education and genetic risks on hippocampal structure in Alzheimer’s disease patients. Patients carrying the APOE4 risk allele and having a first-degree family history of Alzheimer’s disease as well as homozygous APOE4 allele carriers showed a thinner cortex in six of the seven medial temporal subregions, including hippocampal cornu ammonis fields 2, 3, and the dentate gyrus. Patients heterozygous for APOE4 or non-carriers of the risk allele having a first-degree family history of Alzheimer’s disease demonstrated this association only in the parahippocampal cortex. Among subjects without APOE4 or family history risk there was no association between the years of formal education and brain structure.

Environmental factors may account for about half of the variation in Alzheimer’s disease susceptibility in late life [5]. Higher education is a protective factor for the development of Alzheimer’s disease, and has been used as a proxy measure for cognitive reserve [8, 31]. Gatz and colleagues [32] showed that the association remains when accounting for vascular risk factors, socioeconomic status, and other confounders. However, it may be important to consider the effects of a protective factor in the light of a given genetic background. Wang and colleagues [33] found an interaction between APOE4 and educational status for dementia risk. The authors compared dementia risk in 3436 subjects aged 65 and older and showed that high education may buffer the negative effect of APOE4 [33]. Vermeiren and colleagues [21] also demonstrated an interaction of education and the APOE4 risk allele. APOE4 was associated with Alzheimer’s disease only among middle and higher educated subjects. The authors hypothesize that competing risks, such as unhealthier diets and living in an adverse and stressful environment, may be more prevalent in low educated subjects, reducing the contribution of APOE4 in a risk factor pattern [21]. In another study, APOE4 did not modify the association between education and dementia risk, but the risk was particularly low among APOE4 non-carriers with high education [34]. The authors conclude that the association between low education and dementia is not explained by unhealthy lifestyles [34]. These data point to a gene-environment interaction for APOE4 and education in determining dementia risk. The complexity of known and unknown risks in a population, and the availability or unavailability of information on major risk factors, such as a first-degree family history of Alzheimer’s disease, may contribute to the heterogeneous findings in the literature.

In our study the influence of education on cortical thickness was detectable only among patients carrying the highest risk profile. This could suggest a protective mechanism, counteracting the genetic risks’ effects on brain structure that have been demonstrated in cognitively healthy people [20]. In a recent study, small left entorhinal cortex volumes predicted the onset of mild cognitive impairment only in cognitively healthy individuals with lower cognitive reserve [35]. Genetic risk-associated cortical thinning in key regions susceptible to neurodegenerative changes may represent a neuroanatomical feature that occurs early in brain development [36]. However, our data show that the interaction between genetic risks and education with respect to cortical thickness is not restricted to the entorhinal cortex or to the hippocampus, where Alzheimer’s disease pathology would be detectable earlier in the course of the disease than in other regions [2]. It is also not restricted to medial temporal lobe regions, the entorhinal cortex and the subiculum, in which APOE4 genetic risk contributes to cortical thinning among cognitively healthy people [17]. Although we found an interaction effect in these regions as well, we also detected robust interaction effects in the perirhinal and parahippocampal cortices. This suggests that how education and genetic risks interact in their influence on cortical thickness could be a less localized phenomenon compared with APOE4 effects on cortical morphology, although it would have to be investigated using other MRI data analysis techniques for cortical thickness measurements across the whole brain. Others previously demonstrated the influence of education on cortical thickness or volumetric measures outside the medial temporal lobe [12, 13], but these studies did not investigate a possible interaction with genetic risks. Although high-resolution MRI alone cannot be used to differentiate etiologies of cortical thinning, our data suggest that cortical thickness in the hippocampal area is not static, even in demented individuals. Theoretical models of cognitive reserve imply not only active processes our brain can use to cope with neuronal damage, such as network efficiency or compensatory neuronal networks [7], but also greater neuronal count or synaptic density [37] that render a region less susceptible to damage.

Greater cognitive reserve may be associated with accelerated cognitive decline among higher educated patients after the onset of clinical symptoms due to a “threshold effect” with higher education initially masking the clinical manifestation of dementia [8, 31]. The cognitive reserve hypothesis also suggests that higher or lower educated patients do not differ in the age of disease onset, and that higher educated subjects perform better in baseline neurocognitive assessments [8]. Our data are in line with these findings, although the study was not primarily aimed at investigating neurocognitive performance.

This study has several limitations. The imaging approach we use is specifically aimed at investigating subregional hippocampal and medial temporal lobe thickness. With this technique we cannot measure cortical thickness across the whole brain. It remains to be determined whether the interaction of education and genetic risks would also influence cortical thickness in other regions. Education is a reliable proxy measure for cognitive reserve, but the influence of other parameters, such as IQ, the history of occupational work complexity, or social integration could have influenced the results. Furthermore, it is not possible to determine whether APOE4 subjects with a family history carry two separate risk factors according to our model, since these patients could have a family history because of the APOE4 allele. However, then we would have allocated intermediate risk subjects into our high-risk group, which suggests that we could have missed even more pronounced effects on cortical thickness. Family history risk is conceptualized as a heterogeneous factor that can be used to investigate the effects of known and unknown genetic risks [18]. The individual susceptibility genes contributing to the family history could vary across subjects, and even non-genetic factors that are passed to the next generation, such as socioeconomic status [38], could also be part of the composite risk factor [18]. Again, heterogeneity within a risk factor could be associated with an underestimation of effects. We cannot yet determine whether our findings are specific for Alzheimer’s disease patients. Our previous data in healthy people [20] do not suggest comparable associations. However, the number of cognitively healthy subjects we previously investigated is smaller, which could have prevented the detection of similar findings. In addition, these subjects were younger on average, and the influence of protective variables could become more important with increasing age [5]. Finally, whether the influence of education on neuropsychological outcomes would be modulated by genetic risk factors as well, would have to be investigated separately in a larger cohort of individuals differing in cognitive abilities.

The utilization of high-resolution MRI and cortical unfolding image analysis can account for inter-individual anatomical variability as well as for atrophy-associated changes in patients with Alzheimer’s disease. In our study this approach yielded insight into how local brain structure is modulated by an interaction of genetic risks and education. Cortical thickness in the hippocampal region may underlie dynamic changes associated with cognitive reserve, which could influence the effects of genetic risks and neurodegeneration.
